# Chasing weakly-bound biological water in aqueous environment near the peptide backbone by ultrafast 2D infrared spectroscopy

**DOI:** 10.1038/s42004-024-01170-x

**Published:** 2024-04-11

**Authors:** Juan Zhao, Pengyun Yu, Tiantian Dong, Yanzhou Wu, Fan Yang, Jianping Wang

**Affiliations:** 1 Molecular Reaction Dynamics Laboratory, CAS Research/Education Center for Excellence in Molecular Sciences, Institute of Chemistry, Chinese Academy of Sciences, Beijing, 100190 China; 2 University of Chinese Academy of Sciences, Beijing, 100049 China

**Keywords:** Physical chemistry, Biophysical chemistry, Structural biology, Proteins, Infrared spectroscopy

## Abstract

There has been a long-standing debate as to how many hydrogen bonds a peptide backbone amide can form in aqueous solution. Hydrogen-bonding structural dynamics of *N*-ethylpropionamide (a β-peptide model) in water was examined using infrared (IR) spectroscopy. Two amide-I sub bands arise mainly from amide C=O group that forms strong H-bonds with solvent water molecules (SHB state), and minorly from that involving one weak H-bond with water (WHB state). This picture is supported by molecular dynamics simulations and ab-initio calculations. Further, thermodynamics and kinetics of the SHB and WHB species were examined mainly by chemical-exchange two-dimensional IR spectroscopy, yielding an activation energy for the SHB-to-WHB exchange of 13.25 ± 0.52 kJ mol^‒1^, which occurs in half picosecond at room temperature. Our results provided experimental evidence of an unstable water molecule near peptide backbone, allowing us to gain more insights into the dynamics of the protein backbone hydration.

## Abstract (web summary)

Water at the surface of proteins is known to be critically important in maintaining their conformation and function, but the dynamics of the backbone hydration of peptides remains somewhat elusive. Here, the authors study the hydrogen-bonding structural dynamics of *N*-ethylpropionamide, a *β*-peptide model, in heavy water using nonlinear infrared spectroscopy and MD simulations.

## Introduction

Biological water at the surface of proteins is known to be critically important in maintaining their conformations and functions^1–5^. In aqueous solutions, a protein backbone forms hydrogen bonds with solvent water molecule through amide unit (‒CONH‒)^6–11^, which plays an important role in protein hydration. Here, one C=O group can statistically form at most two hydrogen bonds if geometrically allowed, whereas one N‒H group can only form one hydrogen bond^6–10,12^. A representative study^13^ of the hydration of α-helix in a set of 35 high-resolution X-ray crystal protein structures revealed three typical types of hydrogen-bonding interactions between peptide backbone and water molecule: external hydration, three-centered hydration, and water inserted hydration. The external hydration is the most common case where a peptide backbone amide C=O of the *i*th residue that is hydrogen bonded to the amide N‒H group of the (*i* + 4)th residue forms a hydrogen bond with a nearby water molecule. In the three-centered hydration case, the C=O and N‒H groups in an intramolecular (C=O)_*i*_^**…**^(N‒H)_*i+*4_ hydrogen bond forms two hydrogen bonds with the same nearby water molecule. In the water inserted hydration, the (C=O)_i_ group interacts with the (N‒H)_i+4_ groups through a water molecule as a hydrogen-bonded bridge. While the above study provided an averaged structural description of the peptide backbone hydration, the dynamical picture of such hydration particularly in aqueous solutions is still missing, because it is experimentally difficult to track the backbone-associated mobile water molecule in the presence of bulk water.

Among many experimental^14–17^ and computational^18,19^ techniques used to study the protein hydration, infrared (IR) spectroscopy is known to be extremely structure sensitive^20–25^. While the vibration frequency and line shape of amide-I vibration (mainly the C=O stretching motion) are both sensitive to hydrogen-bonding interaction between peptide backbone and solvent water, they are also responsive to electrostatic interactions among neighboring peptide units^26–29^. Therefore, to focus on the amide-water interaction and ignore the intra-peptide interaction contributions, it is ideal to use shorter peptides. N-methylacetamide (NMA) popularly serves as α-peptide model with a single amide unit, and very interestingly, the amide-I absorption band of NMA in water was found to have an inhomogeneously broadened and slightly asymmetric absorption band^30–32^, suggesting a two-component band structure. However, the origin of such asymmetric lineshape remained ambiguous: it was interpreted as the result of vibrational coupling between the amide-I and water bending modes (in H_2_O)^30^, or the result of Fermi resonance (in D_2_O)^31^.

Femtosecond two-dimensional infrared (2D IR) spectroscopy developed in recent years can be used to address this issue, because it is good at revealing dynamical information underneath broad and/or overlapped IR absorption peaks. This method has been used to describe local conformation dynamics of peptides and proteins using the amide-I mode as an intrinsic IR probe^33–40^. In particular, Hamm et al. reported a larger frequency split in the amide-I mode of NMA in methanol and observed picosecond chemical exchange between two solvated NMA/methanol complexes using the 2D IR spectroscopy^41^.

**Fig. 1:**
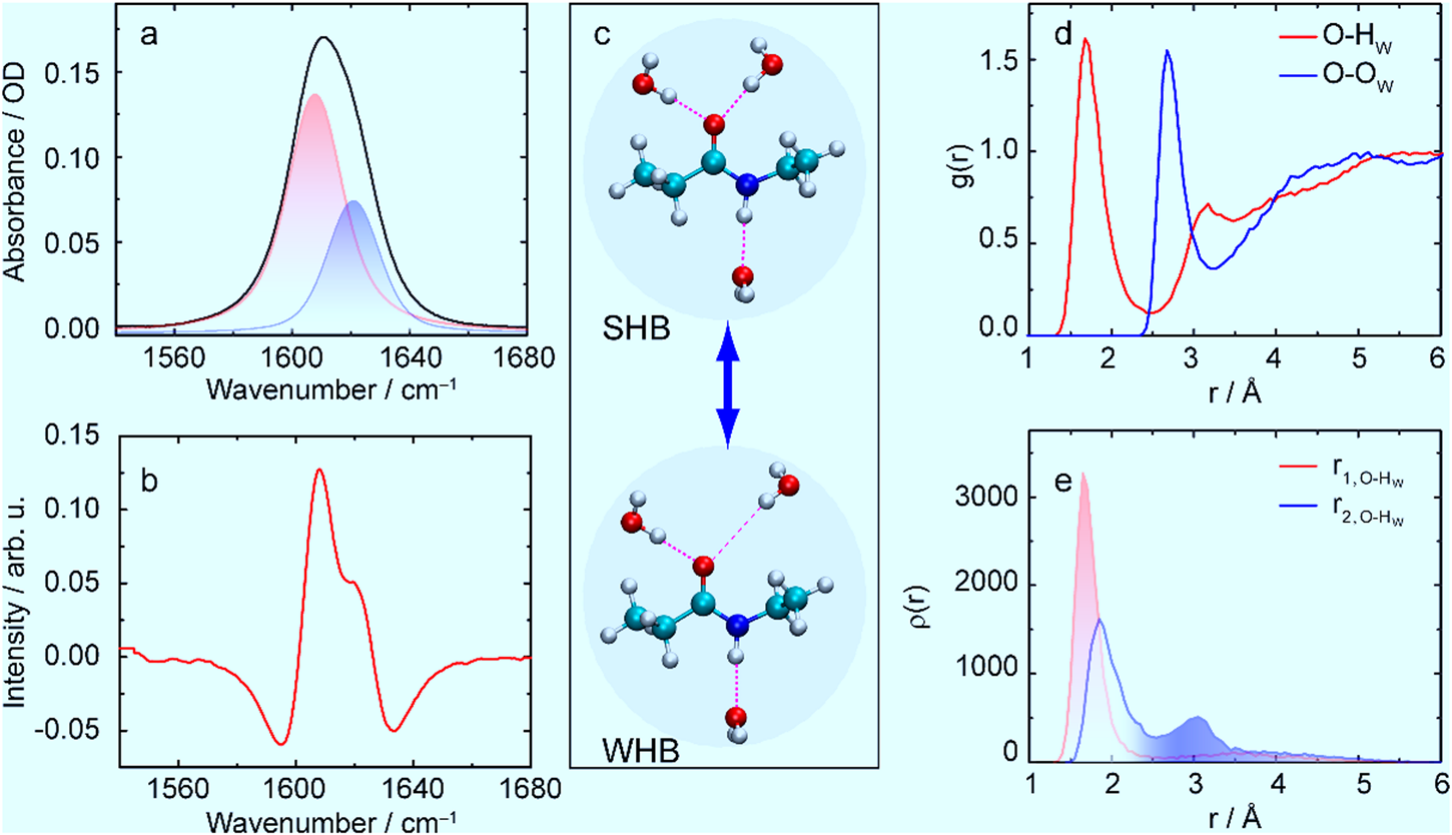
Infrared spectra, chemical exchange, and radial distribution functions. **a** Infrared spectrum of the amide-I band of deuterated *N*-ethylpropionamide (NEPA) in D_2_O at 23 °C with Voigt peak fitting (filled color). **b** Second-derivative IR spectrum to show the presence of two amide-I components. **c** *Trans*-NEPA and water complexes in the strongly hydrogen-bonded (SHB) state and weakly hydrogen-bonded (WHB) state. Chemical exchange occurs between the two states by forming and breaking/weakening one of the amide C = O and water hydrogen bonds. **d** Radial distribution function of water H atom (H_W_) around NEPA carbonyl O atom (red), and that of water O atom (O_W_) around NEPA carbonyl O atom (blue). **e** Statistic probability of the nearest distance $({r}_{1,{{{{\rm{O}}}}}-{{{{{\rm{H}}}}}}_{{{{{\rm{W}}}}}}})$ and the second nearest distance $({r}_{2,{{{{\rm{O}}}}}-{{{{{\rm{H}}}}}}_{{{{{\rm{W}}}}}}})$ between H_W_ and the NEPA carbonyl O atom.

In this work, we used a molecule named NEPA (N-ethylpropionamide) introduced by Shi et al. ^42^ as a single amide model for β-peptide to study the amide-water interaction. The β-peptide is composed of β-amino acid residues and is known to be one of the important unnatural peptides^43–46^. The presence of one extra backbone carbon in the β-peptide results in more variation in the amide-I absorption profile and in the distribution of nearby biological water molecules as well. For NEPA, the amide-I mode in D_2_O is found to be clearly asymmetric and has two spectral components (Fig. 1a, b). Using the 2D IR approach, we studied the origin of the band split. Further, temperature-dependent Fourier-transform IR (FTIR) and 2D IR studies were carried out to characterize the thermodynamics of the hydrogen-bonded NEPA-D_2_O complexes and to obtain the energetic parameters of such hydrogen-bonded complexes. The temperature dependence of the water-amide hydrogen-bonding interaction of the α-peptide reported earlier^47^ was also similarly seen in NEPA, and the result was in agreement with previous studies using the vibrational transition frequency and intensity of the amide-I band^21,32,48^. Hence the analogy between NEPA and the α-peptide in the study of amide hydration is justified. Based on this, the reported monotonic frequency shift of ca. +0.07 cm^–1^/°C and intensity decrease of the amide-I mode with temperature from early works of Kubelka et al. for both α-oligopeptide and NMA^32,49^ were used as an important benchmark for the data analysis of NEPA in this work. By carrying out our study, we were able to capture the motion of a nearby water molecule that is weakly hydrogen bonded with the amide C=O.

## Results and discussion

### FTIR signature of the NEPA/water complexes

Infrared spectrum of the amide-I band of deuterated NEPA in D_2_O at room temperature (23 °C) is shown in Fig. 1a. D_2_O instead of H_2_O is used to avoid interference of HOH bending mode and the amide-I mode of NEPA. The spectrum exhibits an asymmetric absorption peak at 1610.6 cm^–1^ with a shoulder located on the high-frequency side. Its second-derivative spectrum (Fig. 1b) clearly reveals two sub bands, allowing the spectrum in Fig. 1a to be reasonably fitted by two Voigt functions, whose peak positions are at ca. 1607.8 and 1620.9 cm^–1^ respectively. The frequency difference between the two sub bands (Δω = 13.1 cm^–1^) is smaller than that between a typical hydrogen-bonded amide-I mode and its HB-free species as shown by a previous computational work (ca. 20 cm^–1^)^50^. Also, for a single peptide unit such as NMA, the concentration for aggregation in water is known to be about 6 M^41^. Thus, the band splitting cannot be simply due to fully-hydrated (or aggregated) amide species and hydrogen-bond free amide species. Further, the two sub bands are unlikely related to the coupling between the amide-I mode and water bending mode either, because deuterated water is used specifically as solvent in our work, and DOD bending frequency is below 1300 cm^–1^ ^51^.

The inhomogeneously broadened amide-I band of deuterated NMA in D_2_O was also symmetric and was believed to be caused by Fermi resonance^31^. For NEPA studied in this work, the two sub bands are unlikely due to Fermi resonance for two reasons. First, there are also two sub bands for the amide-I band of non-deuterated NEPA in H_2_O (see Supplementary Fig. 1). The intensity ratio of the two sub bands for non-deuterated NEPA in D_2_O is 2.136:1 (the low-frequency component vs. the high-frequency one), which is generally consistent with that of deuterated NEPA in D_2_O (2.260:1). Second, quantum-chemistry (QC) calculations using the second-order vibrational perturbation theory (VPT2)^52^ indicate that there is no combination or overtone band resonating with the amide-I band of NEPA in explicit solvent (NEPA‒3D_2_O cluster) or in implicit D_2_O solvent using the polarizable continuum model^53,54^.

The possibility of two different conformations (*trans* and *cis* amide) of NEPA^42^ can also be ruled out because the ^1^H and ^13^C NMR spectra of NEPA in water (Supplementary Data 1) showed only one conformation of NEPA. In addition, the QC calculations indicate that the energy of the *trans* conformation is 8.42 kJ mol^‒1^ lower than the *cis* conformation, suggesting that the *trans* NEPA is the dominant structure in the gas phase and presumably be so in the aqueous phase.

### Simulation reveals structural details of the NEPA/water complexes

Next, molecular dynamics (MD) simulations and QC calculations were carried out to investigate the origin of the two sub bands of the amide-I mode of NEPA in D_2_O. Fig. 1d shows the radial distribution function (RDF) of water H atom (H_W_) around carbonyl oxygen of NEPA. The maximum peak appears at 1.65 Å and the value of the first RDF valley appears at 2.55 Å; the latter indicates the maximum distance between H_W_ and O atom of NEPA in the first hydration shell. The number of H_W_ is found to be 2.06 on average in this hydration shell, determined using a previously described method^55^. However, the number of water O atom around amino H atom of NEPA is close to 1.0, indicating approximately one hydrogen-bonded water in the neighborhood of the N‒H group.

Figure 1e shows the statistics of the two nearest distances between the carbonyl O atom and H_W_, based on time-dependent MD trajectories up to 5 ns (Supplementary Fig. 2). There is only one peak in the nearest distance $({r}_{1,{{{{\rm{O}}}}}-{{{{{\rm{H}}}}}}_{{{{{\rm{W}}}}}}})$, which is located at 1.65 Å. However, two peaks are shown in the second nearest distance $({r}_{2,{{{{\rm{O}}}}}-{{{{{\rm{H}}}}}}_{{{{{\rm{W}}}}}}})$, which are peaked at 1.85 and 3.0 Å respectively. Further, according to a well-used hydrogen bond criterion^56^, *i.e*., the donor-acceptor distance is less than 3.5 Å and the donor-proton-acceptor angle is less than 40°, averaged number of hydrogen bonds formed between the C=O group of NEPA and water is found to be ca. 1.39 from our MD results. The first valley of the RDF of water oxygen atom around carbonyl oxygen appeared at 3.25 Å (Fig. 1d), falling into the range of the hydrogen-bonding criterion (3.5 Å). The statistic distance and the hydrogen-bond numbers indicate that at a given dynamical time at least one strong hydrogen bond is formed in the hydration shell of the C=O group of the NEPA. Furthermore, the two-peak profile of ${r}_{2,{{{{\rm{O}}}}}-{{{{{\rm{H}}}}}}_{{{{{\rm{W}}}}}}}$ suggests a time-dependent bonding strength and probability for the second hydrogen bond between the C=O group and a water molecule. Thus, the MD results suggest that there are two differently solvated amide C=O groups of NEPA in D_2_O, i.e., the C=O group can either form two strong hydrogen bonds with the surrounding water molecules in the hydration layer (strong hydrogen-bonding state, SHB), or form one strong and one weak hydrogen bond between C=O and water (weak hydrogen-bonding state, WHB).

In addition, the distance between the amino H atom and water O atom only shows one broad peak for its statistic distribution (Supplementary Fig. 3). Density functional theory (DFT) calculations show that the frequency shift caused by the hydrogen bond formed on the N‒H site is smaller than that formed on the C=O group^50^. Therefore, the contribution of the dynamical water hydrogen bond on the N‒H side is unlikely to be the reason for the observed amide-I doublet.

Taken together, we believe the two-sub band profile of the amide-I band of *trans*-NEPA in D_2_O is associated with the heterogeneous distribution of the hydrogen bonds formed on the C = O side. Particularly, it is due to the effect of a weak hydrogen bond from a nearby water on the amide-I frequency. The weakening or breaking of one of the C=O/D_2_O hydrogen bonds (Fig. 1c bottom) gives rise to the high-frequency amide-I component, while the low-frequency component (1607.8 cm^–1^) is the dominant component associated with the majority of the amide groups in NEPA molecules that is fully hydrated (containing three hydrogen bonds, Fig. 1c top). Our QC calculation also indicates that the amide-I frequency of NEPA in the SHB state (Fig. 1c top) is about 14 cm^–1^ lower than that in the WHB state (Fig. 1c bottom), which agrees reasonably with the experimentally observed frequency difference (13.1 cm^–1^) between the two sub bands of amide-I band in Fig. 1.

### Thermal behavior of the NEPA/water complexes

**Fig. 2:**
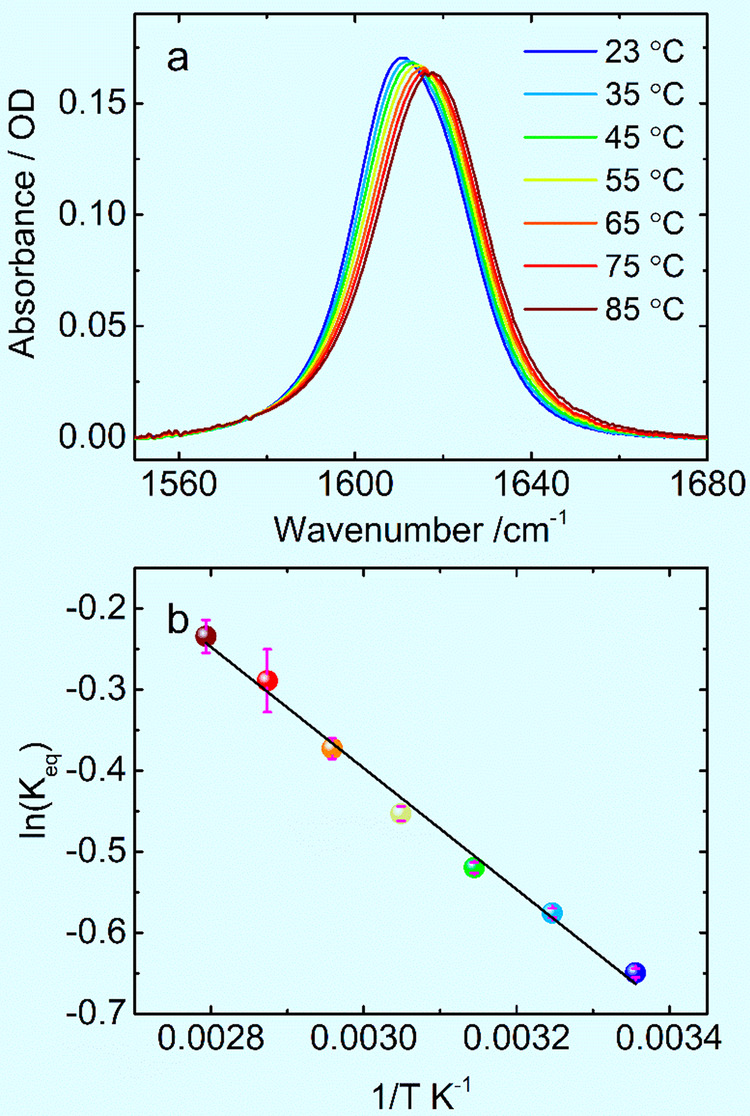
Temperature-dependent amide-I infrared spectra and derived van’t Hoff plot. **a** Temperature-dependent infrared spectra of the amide-I band of deuterated *N*-ethylpropionamide (NEPA) in D_2_O from 23 to 85 °C at 10 °C interval. **b** The van’t Hoff plot using the equilibrium constant of the weakly hydrogen-bonded (WHB) state and strongly hydrogen-bonded (SHB) state populations as a function of temperature. See Supplementary Note 1 for error bars’ generation.

Temperature-dependent FTIR spectra of NEPA in D_2_O were measured to investigate the thermal stability of the NEPA/water complexes illustrated in Fig. 1c. The results are shown in Fig. 2a. First, Fig. 2a shows that the overall intensity of the amide-I band decreases slightly as temperature increases, which is due to the decrease of the molar extinction coefficient of the amide-I mode as a function of temperature. This may also be partially related to the temperature-dependent solute molecular cavity, as well as temperature-dependent dielectric constant and refractive index of solvent^32^. Second, similar to the room-temperature result, the infrared spectra at elevated temperatures remain to be asymmetric.

The second derivative spectra also show that there are generally two main peaks for the amide-I band as temperature changes, and the low-frequency peak blue shifts clearly with increasing temperature, while the high-frequency peak only slightly shifts (Supplementary Fig. 4), reflects the dynamical nature of the structure complexes giving rise to these two amide-I components. The fitting spectra are shown in Supplementary Fig. 5 with fitting parameters listed in Supplementary Table 1. The obtained peak position of the low-frequency component varies with temperature at a rate of 0.07 cm^–1^/°C), agreeing with the result of a previous work where the amide-I band of NMA and a α-oligopeptide both exhibit a linear frequency dependence with temperature^49^. Thus, the low-frequency peak in Fig. 1a represents the well-hydrated amide unit in NEPA, while the high-frequency peak represents the WHB configuration.

Furthermore, as temperature increases, the peak area of the SHB state decreases, while that of the WHB state increases (Supplementary Fig. 5). In the IR spectroscopy, the integrated absorption peak is proportional to *C*_*i*_|*μ*_*i*_|^2^, where *C*_*i*_ and |*μ*_*i*_| respectively represent the molar fraction (or concentration) and transition dipole moment magnitude of the amide-I mode in a given hydrogen-bonded state (*i* stands for SHB or WHB). If the ratio of the transition dipole moment in the two states was assumed to be temperature independent, then the decreased peak area ratio of the SHB state to the WHB state suggests a decreased NEPA population of the SHB state and an increased population of the WHB state. Thus, as temperature increases, the interaction between NEPA and water becomes weaker and some of the NEPA molecules in the SHB state turned into those in the WHB state. This suggests a temperature-induced dehydration for the amide group of NEPA. Hence the ratio of the integrated IR absorption area in the WHB state (*A*_WHB_) and that in the SHB state (*A*_SHB_) yields the ratio of concentrations of NEPA in the WHB and in SHB states, which in turn leads to the equilibrium constant *K*_eq_ for the WHB formation at a given temperature:

1 $${K}_{{{{{\rm{eq}}}}}}=\frac{{C}_{\left[{{{{\rm{WHB}}}}}\right]}}{{C}_{\left[{{{{\rm{SHB}}}}}\right]}}=\frac{{A}_{{{{{\rm{WHB}}}}}}\times {\left|\mu \right|}_{{{{{\rm{SHB}}}}}}^{2}}{{A}_{{{{{\rm{SHB}}}}}}\times {\left|\mu \right|}_{{{{{\rm{WHB}}}}}}^{2}}$$

Here, the transition dipole of the amide-I mode in each state (Fig. 2a) is evaluated by the QC calculation in the gas phase, which are 0.344 Debye (*D*) and 0.321 *D* for the SHB state and WHB state respectively. The fitting results of the two sub bands at varying temperature are listed in Supplementary Table 1.

From the temperature-dependent equilibrium constant, a van’t Hoff plot is obtained and shown in Fig. 2b, which yields Δ*H* = 6.22 ± 0.25 kJ mol^–1^ and Δ*S* = 15.34 ± 0.76 J mol^–1^ K^–1^. Δ*H* > 0 indicates an endothermic transition from the SHB state to the WHB state, while Δ*S* > 0 suggests an increased inhomogeneity with temperature. Further, the effect of the transition dipole moment ratio of the two states on the thermodynamic properties was considered. When the C = O group forms one hydrogen bond with one water molecule, which is one of the extreme cases of the WHB state, the transition dipole of the amide-I band is about 0.304 D by the QC computation of this work. The other extreme is that the transition dipole of the amide-I mode in the WHB state is the same as that in the SHB state. The ratio of transition dipole of the amide-I mode in the SHB state verses that in the WHB state in these two extreme cases are therefore 1:1 and 1.32:1, respectively. In these two cases, van’t Hoff plot shows that the Δ*H* (and Δ*S*) are 6.22 kJ mol^–1^ (14.20 J mol^–1^ K^–1^) and 6.22 kJ mol^−1^ (16.25 J mol^–1^ K^–1^). These results illustrate that even though the ratio of the transition dipole of the amide-I mode in the SHB state verses that in the WHB state is different in these conditions, the enthalpy change (Δ*H*) remain unchanged, while the entropy change (Δ*S*) varies to a limited extent, but still falls within its experimentally determined range.

Very recently, the amide-water interaction was also examined based on polymeric units with varying N-alkyl groups including NEPA^57^. Their temperature-dependent IR spectra showed that the enthalpy change and entropy change are 4.8 kJ mol^–1^ and 12 J mol^–1^ K^–1^ respectively, which are close to the values reported in our work. Further, a theoretical estimation yielded the hydration enthalpy of –23 kJ mol^–1^ for the C=O group of NMA with one water molecule^58^. A previous work also showed that the enthalpy change associated with the formation of a hydrogen bond between the C=O group in small peptides and water molecule is in the range of –20 ~ –30 kJ mol^–19,^ ^59–61^. Therefore, the enthalpy change from the SHB state to the WHB state in our work is significantly smaller than the dissociation energy of a hydrogen-bond water from an amide unit, indicating that in the WHB state studied here, the second hydrogen bond formed is definitely weaker than the fully hydrated amide group.

### 2D IR signature of chemical exchange between the two NEPA/water complexes

**Fig. 3:**
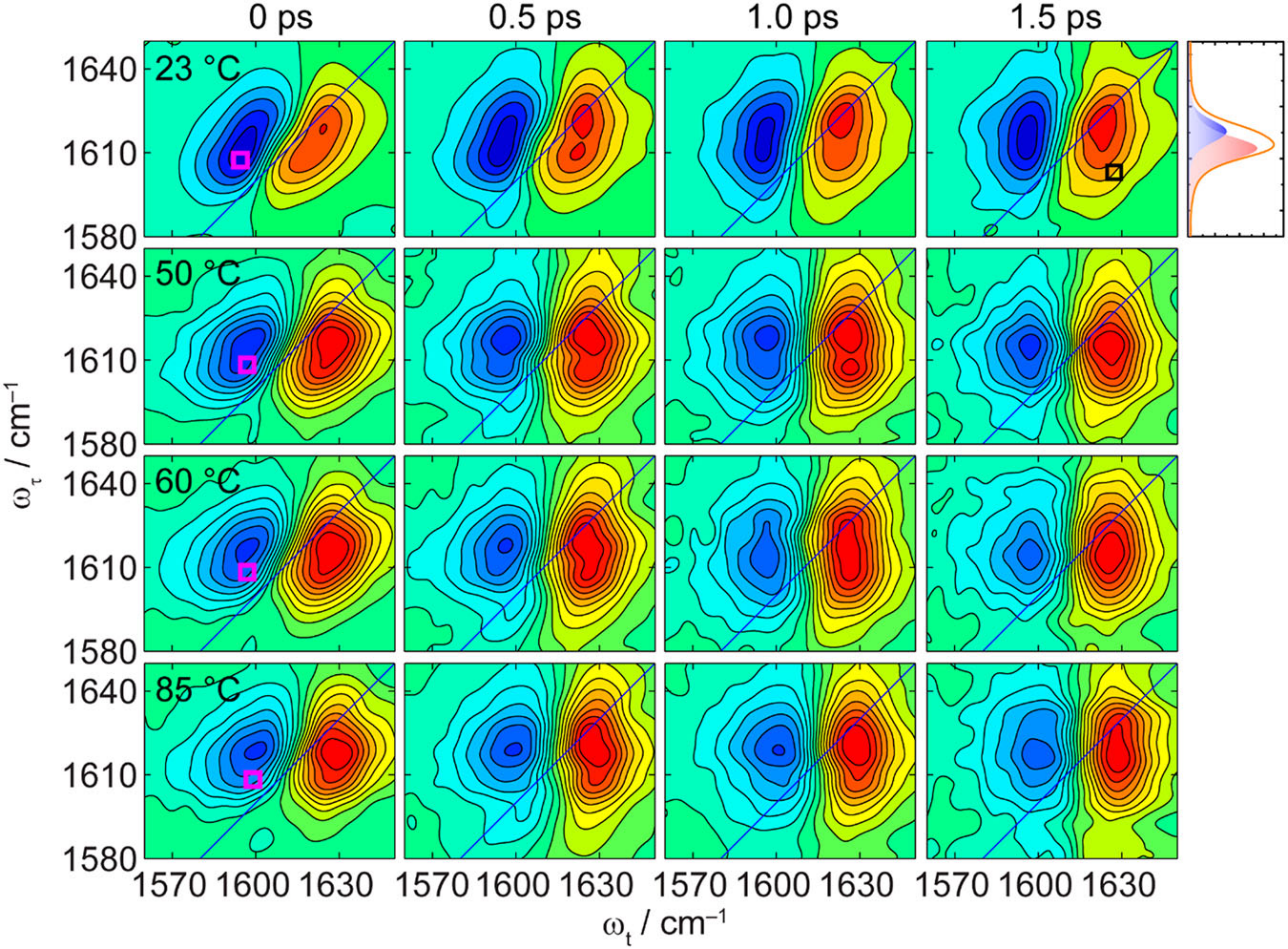
Waiting-time dependent, purely absorptive 2D IR spectra of NEPA in D_2_O in the amide-I region at different temperatures. The FTIR spectrum at 23 °C with two-component fitting is shown in the upper right panel. Pink squares in left panels mark the diagonal peak area for integration (ω_τ_ = 1605–1610 cm^–1^ and ω_t_ = 1592–1597 cm^–1^ at 23 °C; ω_τ_ = 1606–1611 cm^–1^ and ω_t_ = 1594–1599 cm^–1^ at 50 °C; ω_τ_ = 1606–1611 cm^–1^ and ω_t_ = 1594–1599 cm^–1^ at 60 °C; ω_τ_ = 1606–1611 cm^–1^ and ω_t_ = 1597–1602 cm^–1^ at 85 °C). A black square in an area of ca. 25 cm^–2^ in upper right panel indicates the region of the cross peak to be extracted, after subtracting the spectrum at 0 ps, for the examination of the chemical exchange dynamics from the strongly hydrogen-bonded (SHB) state to the weakly hydrogen-bonded (WHB) state.

Figure 3 shows purely absorptive 2D IR spectra of NEPA in D_2_O in the amide-I region at varying temperature (23 °C, 50 °C, 60 °C and 85 °C) at a few selected waiting times (*T*_w_). More sets of spectra are given in the Supplementary Figs. 6–9. The corresponding FTIR spectrum at 23 °C is shown on the upper right corner of the figure. Red (positive) 2D IR peak is the *v* = 0 to *v* = 1 (0 → 1) transition, where *v* is the vibrational quantum number, and the corresponding blue (negative) peak on the left side is the 1 → 2 transition. The *ω*_t_ frequency of the latter is lower than that of the former because the amide-I vibration is anharmonic. The 2D IR spectra show that there are two diagonal red peaks in the amide-I region, which is more obvious at longer waiting time and higher temperature, being consistent with the presence of the two hydration states of the amide group of NEPA discussed above. Further, as the waiting time increases, two pair-wise and partially overlapped cross peaks between these two diagonal peaks appear at the anti-diagonal direction (i.e., on the upper left and lower right corners of a 2D plot shown in each panel of Fig. 3).

**Fig. 4:**
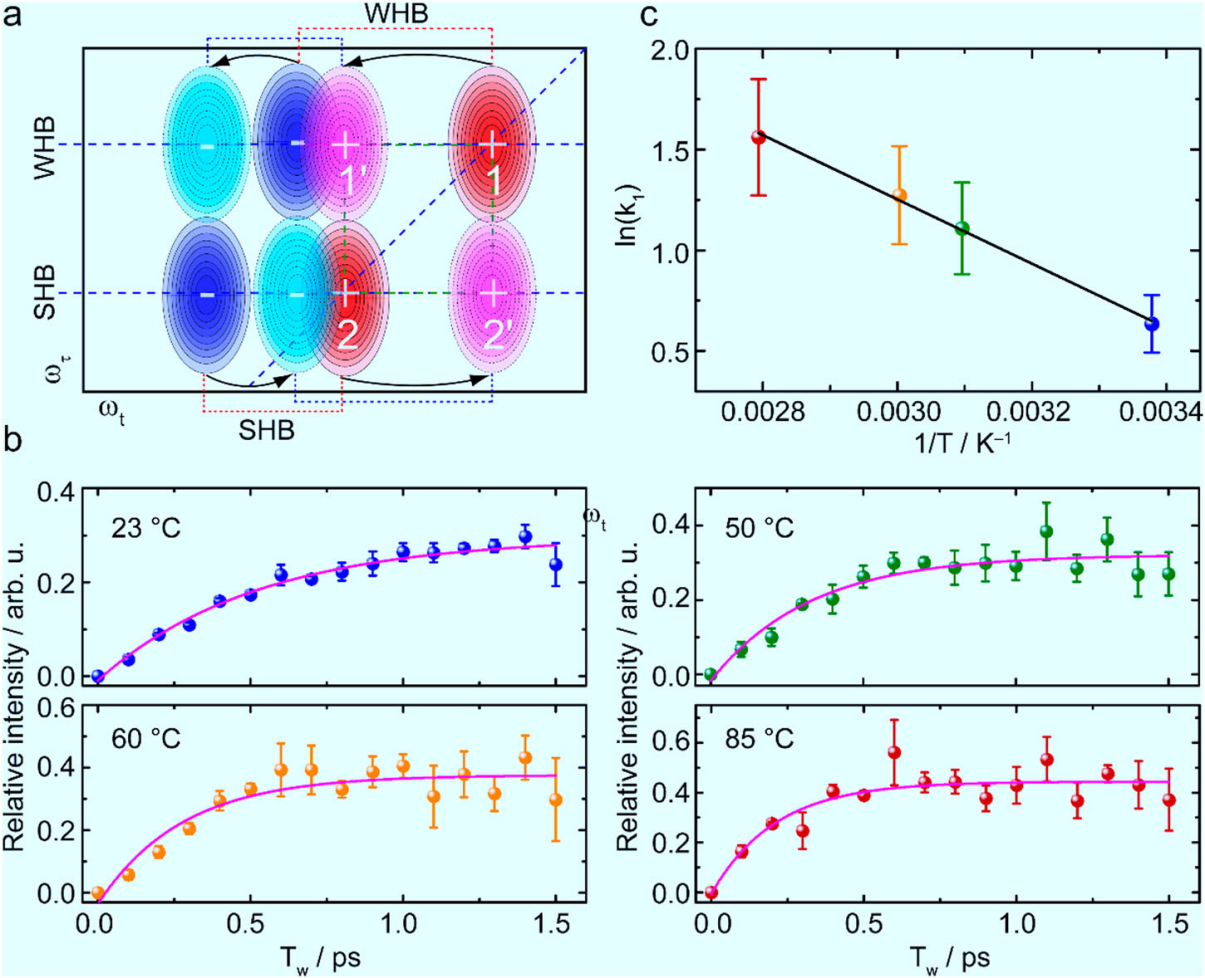
Chemical-exchange 2D IR and the exchange dynamics. **a** An illustration of a typical 2D IR spectrum of the two-component amide-I mode of *N*-ethylpropionamide (NEPA) at longer waiting time (*T*_w_) in the presence of chemical exchange (more description is given in Supplementary Fig. 10). **b** Time evolution of the relative magnitude (dots) of the 2D IR cross peak and its fitting by a signal exponential function (solid line) at each temperature. The obtained exchange rate constant from the strongly hydrogen-bonded (SHB) state to the weakly hydrogen-bonded (WHB) state is 1/(0.53 ± 0.07) ps^‒1^, 1/(0.33 ± 0.07) ps^‒1^, 1/(0.28 ± 0.07) ps^‒1^, and 1/ (0.21 ± 0.06) ps^‒1^ at 23 °C, 50 °C, 60 °C, and 85 °C respectively. **c** The Arrhenius plot of the rate constant from the SHB state to the WHB state versus temperature with a linear fitting. Error bars were produced by analyzing repeating 2D IR experimental measurements.

The possibility of these cross peaks due to intermolecular coupling or due to intermolecular vibrational energy transfer, assuming the SHB and WHB species of NEPA shown in Fig. 1c are independent in time, is unlikely at the concentration of 100 mM. Chemical exchange between the SHB and WHB species is the only source of these cross peaks. The phenomenon is very similar to the chemical exchange reported within dynamical NMA-methanol complexes by the 2D IR method^41^. This suggests that the hydrogen-bonded NEPA/water complexes are very dynamic and the nearby water molecules can leave NEPA with a weakened or broken hydrogen bond, so that the cross-peak pair in the lower-right region of a 2D IR spectrum comes from the chemical exchange from the low-frequency vibrator (representing the SHB state) to the high-frequency vibrator (representing the WHB state), whereas the cross peak pair in the upper-left region is due to the reverse process. This is illustrated in Fig. 4a briefly with more description given in Supplementary Fig. 10. Considering the limited frequency difference between the two amide-I sub bands (13.1 cm^–1^, Fig. 1), the blue peak of the cross-peak pair caused by the exchange process from the low-frequency state to the high-frequency state, and the red peak of the cross-peak pair caused by the reverse exchange process are not spectrally resolved.

It is known that vibrational relaxation, orientational relaxation and spectral diffusion all play a role in influencing the apparent lineshape and signal magnitude of a 2D IR spectrum. In a simplified picture, for a chemically exchangeable system, as *T*_w_ increases, the exchange between the two chemical species causes their diagonal 2D IR peak intensities to decrease and their off-diagonal 2D IR peak intensities to increase^62^, while vibrational relaxation and orientational relaxation both decrease the diagonal and off-diagonal peak intensities. The spectral diffusion changes the shapes of the 2D IR peaks but preserves their total volumes. Given these considerations, the chemical exchange dynamics between the SHB and WHB states can be extracted from the relative growth of the cross-peak intensity as a function of *T*_w_. To evaluate the off-diagonal spectral components while eliminating the effect of the vibrational population and orientational relaxations, each 2D IR spectrum was first normalized using its absolute magnitude with respect to an integrated blue-peak area of ca. 25 cm^–2^ of the low-frequency component (see Fig. 3 left column) representing the 1 → 2 transition of the SHB state (see the cartoon in Fig. 4a for its assignment), then the 2D IR spectrum at *T*_w_ = 0 ps was subtracted from those at various waiting times. then the time evolution of an integrated cross-peak area of 25 cm^–2^ (whose location was shown in Fig. 3 upper right panel, also see Supplementary Fig. 11 for 2D IR difference spectra at three typical waiting times) was used to measure the chemical exchange kinetics from the SHB state to the WHB state, and the results are shown in Fig. 4b at four temperatures using the data presented in the Supplementary Figs. 6–9. A single exponential function was used to fit the chemical exchange process in each case, whose rate constant *k*_1_ is 1/(0.53 ± 0.07) ps^‒1^, 1/(0.33 ± 0.07) ps^‒1^, 1/(0.28 ± 0.07) ps^‒1^and 1/ (0.21 ± 0.06) ps^–1^ at 23 °C, 50 °C, 60 °C, and 85 °C, respectively. The Arrhenius plot based on these rate constants was shown in Fig. 4c, from which an activation energy for the change from the SHB state to the WHB state of *E*_a_ = 13.25 ± 0.52 kJ mol^‒1^ was determined. In addition, the position of the cross peak (demonstrated in Supplementary Fig. 11) along the *ω*_t_ axis slightly shifts to the higher-frequency side as a function of temperature, agreeing with the results shown in Supplementary Fig. 5 and Supplementary Table 1 and showing a dynamical structure nature of the high-frequency component.

**Fig. 5:**
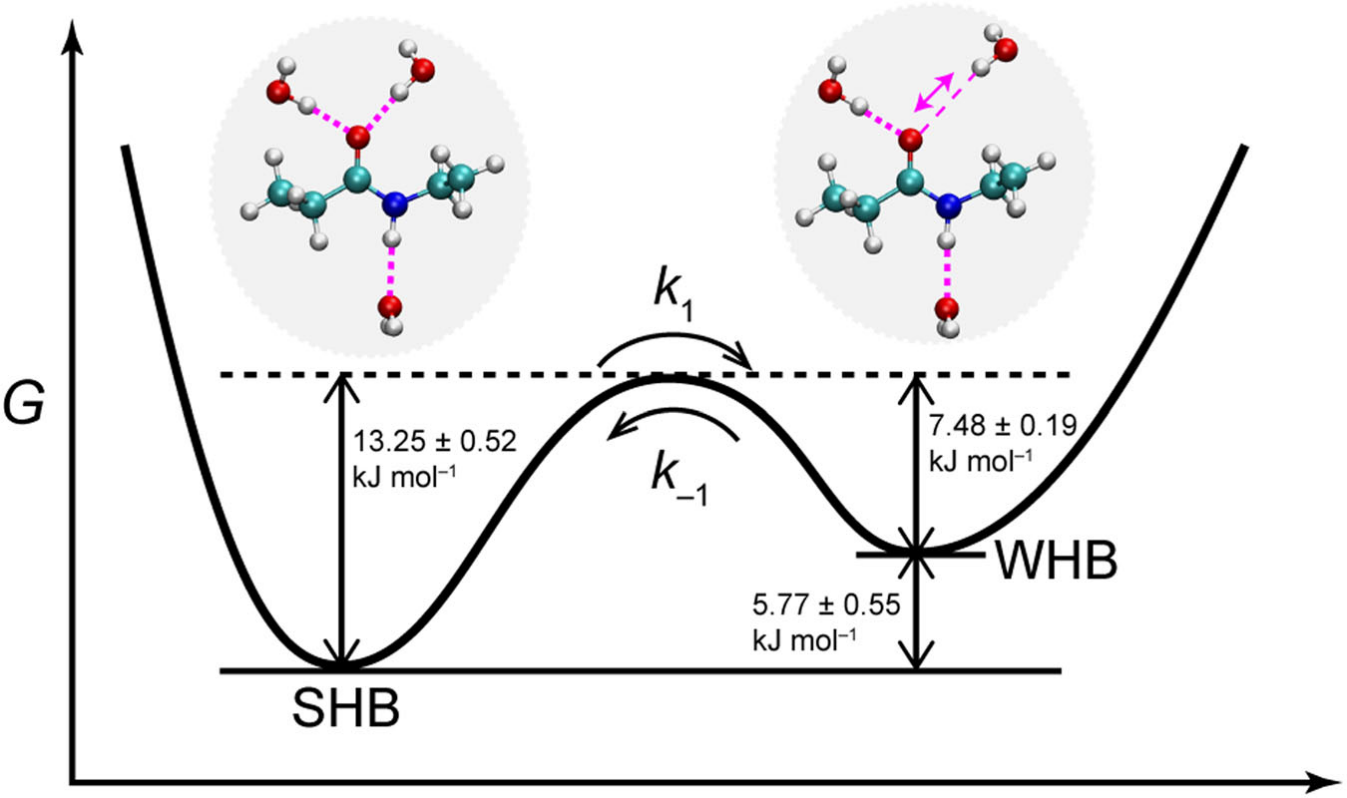
Energy scheme of the hydrated amide unit of *N*-ethylpropionamide (NEPA) in the strongly hydrogen-bonded (SHB) and weakly hydrogen-bonded (WHB) states linked through an amide C=O^…^H_2_O hydrogen bond. Chemical exchange occurs between the two hydration states with their activation energies determined by temperature-dependent 2D IR spectroscopy.

At a given temperature and pressure, according to the van’t Hoff equation $(\Delta G\,=\,\Delta H\,-\,{{{{\rm{T}}}}}\Delta S)$, the free-energy difference ($\Delta G$) between the WHB and the SHB states is found to be 1.68 ± 0.34, 1.27 ± 0.34, 1.11 ± 0.36, and 0.73 ± 0.37 kJ mol^‒1^ at 23 °C, 50 °C, 60 °C, and 85 °C respectively. From the relation ${k}_{1}={k}_{-1}{{{{{\rm{e}}}}}}^{-\Delta G/{k}_{{{{{\rm{B}}}}}}{{{{\rm{T}}}}}}$, where k_B_ is the Boltzmann constant, then the exchange rate *k*_‒1_ (from the WHB state to the SHB state) is roughly 1/0.27 ps^‒1^, 1/0.21 ps^‒1^, 1/0.19 ps^‒1^, and 1/0.16 ps^‒1^ at corresponding temperature. The activation energy for the WHB state to the SHB state obtained from the Arrhenius plot (Supplementary Fig. 12) is 7.48 ± 0.19 kJ mol^‒1^. A direct comparison of *k*_1_ and *k*_‒1_ shows that the exchange process from the WHB state to the SHB state is faster than the reverse process. A typical energy diagram is illustrated in Fig. 5, which shows a schematic representation of the free energy of as a function of the hydrogen bond coordinate for the amide-I mode.

### Backbone hydration for the α- and β-peptides

While the hydration of the β-peptide is much less reported^63^, the backbone and water hydrogen-bonding interaction of the α-peptide has been studied extensively using various methods as mentioned in the beginning of this work. Peptide-water hydrogen bond has been generally known to compete with intrapeptide hydrogen bond^12^, and it was proposed a while ago that peptides and their aqueous environment form a dynamical entity^64^. However, the dynamics of the weakly bound water within such entity was not examined because the unavailability of ultrafast experimental methods during that time. On the other hand, mobile water molecules in the vicinity of backbone C=O and out of the amide plane may form a weak hydrogen bond and red shift the amide-I mode frequency, which has been suggested in Torii’s recent work^26^. Experimentally, Tokmakoff et al. examined the solvation dynamics of dialanine in D_2_O recently^65^, where the chemical exchange dynamics in three solvation structures were studied by the 2D IR spectroscopy. One of the hydration states (state B) where one of the amide C=O is hydrogen-bonded to two solvent water molecules is quite similar to what we describe in this work. However, due to the presence of multiple amide C=O groups, the mobile water in the hydration structure was not traced in that work. On the other hand, the presence of hydrogen-bonded peptide-water molecules has been suggested in a very recent chemical-exchange 2D IR study of model peptides with varying N-alkyl side chains (including NEPA) by Kuroda’s group^57^. Their result showed that there are also two amide-I transitions for these polymeric units in D_2_O, which are generally due to two distinct hydrogen-bonding environments due to amide-water hydrogen making and breaking, but the chemical exchange dynamics of such weakly-bound water was not explicitly examined.

Our detailed analysis in work here demonstrated that NEPA, as a model for a β-peptide unit, shows certain advantage over NMA, which was often used as a model for an α-peptide unit, in a sense that the two amide-I components are slightly more separated (10.0 cm^‒1^ for NMA^31^ and 13.1 cm^‒1^ for NEPA), and hence allows a more convenient study of the 2D IR cross peak dynamics. Our work shows that the two sub bands of the amide-I mode of the peptide unit can be reasonably explained as a result of a dynamical water that switches between weakly and strongly hydrogen-bonded to the amide C=O side, on the basis of a strongly hydrogen-bonded water. Statistically the two bound water molecules may switch their identities, but one of them seems to be always strongly hydrogen bonded. Such situation can be regarded as a simplified picture where the amide C=O group and some nearby N‒H group forms an intramolecular hydrogen bond as commonly seen in secondary structural motifs such as the α-helices and β-sheets. Under such circumstances in the aforementioned external hydration state^13^ determined by high-resolution X-ray crystallography may have actually a dynamical water if the protein is solvated in aqueous solution. Further, vibrational lifetime (*T*_1_) of the amide-I mode for the SHB and WHB states were estimated to be ca. 0.67 ps and 0.72 ps (Supplementary Fig. 13), supporting our proposed dynamical hydration picture because the *T*_1_ process of the amide-I mode reflects the vibrational energy relaxation to solvent water. A slightly shorter one-component time constant (0.45 ps) was reported for NMA in D_2_O probed at the lower frequency side of the SHB state^31^, agreeing in trend with our results of NEPA in D_2_O.

Therefore, the results presented in this work not only fits the generally accepted statistics that peptide backbone amide C=O can form more than one and at most two hydrogen bonds, but also provides new structural and dynamics insights into the weakly hydrogen-bonded water. Our results are also in general agreement with a previous study by DeGrado and coworkers that water molecules are dynamically bonded to amide carbonyl groups in the α-helices with partial occupancy (~50%–70%)^66^.

Further, the influence of water on the IR spectral of the amide-I mode have also been examined by ab initio calculations, MD simulations, as well as empirical modeling (frequency maps based on NMA)^67–72^. However, the two sub-band picture of the amide-I mode in NMA has not been specifically discussed. Thus, our results also call for more detailed computational works. A simple treatment may be to treat amide-water clusters differently out of a MD trajectory, according to hydrogen-bonding strength of the second neighboring water, and apply the frequency maps for the amide-I frequency evaluation. Such work is undergoing in our laboratory based on a previously developed general applicable frequency map for the β-peptide^73^, where an asymmetric amide-I of NEPA in D_2_O was roughly predicted, showing the presence of a weak high-frequency component.

### Conclusions and remarks

In this work, two overlapping amide-I IR components were spectrally resolved for the model β-peptide (NEPA) solvated in deuterated water. IR and NMR spectroscopic measurements, quantum-chemistry calculations and MD simulations together suggest there are statistically two different dynamical hydration states on the amide C=O side of the monopeptide unit: one forms two regular hydrogen bonds with nearby solvent water and yields the so-called strong hydrogen-bonding state in the first hydration shell of the amide group, leading to the low-frequency IR component of the amide I band; the other forms one regular hydrogen bond between C=O and water in the hydration layer and one weak hydrogen bond that may be partially associated with the second solvation layer, which yields the weak hydrogen-bonding state and gives rise to the high-frequency amide-I component.

Time-dependent 2D IR spectra reveal a fast equilibrium chemical exchange between the two solvated states. This exchange process may be common but is hidden underneath the conventional IR spectra of the amide-I mode. The relative magnitudes of the cross peaks at various waiting time reveal the exchange dynamics from the SHB state to the WHB state, whose time constant is ca. 0.53 ps at room temperature. Temperature-dependent 2D IR spectra reveal that the exchange becomes faster as temperature increases. The activation energy of the exchange from the SHB state to the WHB state was determined from the 2D IR spectra to be about 13.25 ± 0.52 kJ mol^‒1^, while that of the reverse exchange was about 7.48 ± 0.19 kJ mol^‒1^. In addition, from the temperature-dependent FTIR results, the enthalpy change from the SHB state to the WHB state is much lower than the earlier obtained dissociation energy of a hydrogen-bond water from an amide C=O group, demonstrating the presence of a weakly hydrogen-bonded water in the NEPA/D_2_O system studied here. In addition, both temperature-dependent FTIR and 2D IR spectra demonstrate a dynamical frequency position of the high-frequency amide-I component, revealing the mobile nature of the weakly bound water molecule.

In summary, our results in this work clearly demonstrate the structural dynamics of a weakly hydrogen-bonded water in the immediate hydration layer of the peptide backbone, which is insightful for understanding the dynamics of the biological water interaction directly with peptide backbone.

## Methods

### Materials

N-ethylpropionamide (NEPA, 99% purity) was purchased from Sigma-Aldrich. It was lyophilized in deuterated water (D_2_O) three times for the H/D exchange of the amide group, and then dissolved as deuterated form in D_2_O at a concentration of 100 mM.

### FTIR spectroscopy

Infrared spectra were collected using Nicolet 6700 FTIR spectrometer equipped with a liquid nitrogen-cooled mercury-cadmium-telluride (MCT) detector. NEPA solution samples were placed in a home-made dual IR cell. The dual IR cell contained two 2-mm thick CaF_2_ windows separated by a 30-*μ*m thick “θ”-shaped Teflon spacer, which forms two independent sample compartments. The dual cell is made of copper, which is heated through a water bath for temperate-depended FTIR measurement. The NEPA/D_2_O solution and pure liquid D_2_O were placed in the two compartments separately. The dual cell was placed on a motorized translation stage so that the sample solution and solvent-only (background) spectra could be taken alternately. D_2_O is used instead of H_2_O to avoid interference of HOH bending mode and amide-I mode of NEPA. Dry air was used to purge FTIR spectrometer and the sample chamber during the IR spectral measurement. FTIR spectra were measured with a spectral resolution of 1 cm^−1^ and averaged by 64 scans from room temperature (23 °C) to 85 °C at 10 °C interval.

### Nonlinear IR spectroscopy

2D IR quick shaper spectrometer (2D Quick, PhaseTech) was used to collect 2D IR spectra^74,75^. A typical laser pulse (3-mJ, sub 25-fs, 800-nm, 1 kHz) was generated using a femtosecond laser system and was used to pump an optical parametric amplifier (OPA) to generate two near-IR pulses, i. e., the signal and idler pulses. The two near-IR pulses were further split by a dichroic mirror and independently controlled in time and collinearly aligned in space, and were loosely focused together on a nonlinear crystal (AgGaS_2_) for difference-frequency generation (DFG), generating a 6-*μ*J mid-IR pulse centered at 6-μm with FWHM of ca. 270 cm^‒1^. The mid-IR pulse was split into pump and probe paths and then spatially and temporally overlapped in the sample. 2D IR signal was generated in the pump-probe geometry, and the polarization of the pump pulse was set as the same condition of the probe pulse. The pump pulse enters an IR pulse shaper (Ge-AOM) and generates a collinear pulse pair with modulated interval time (*τ*). The 2D-IR signal generated by the coincidence of the three pulses on the sample is in the direction of the probe pulse. The waiting time (*T*_w_) between the pump and probe pulses is controlled by a pair of ZnSe wedges. Purely absorptive 2D-IR signal was detected and digitized using an IR monochromator equipped with a 64-element liquid nitrogen-cooled MCT array detector. The sample cell was packaged in the home-made temperature control component composed of a heating plate and a silicone rubber cover, both being connected to an intelligent temperature controller (ZNHW-III), which can maintain the temperature of the sample at a certain value during the 2D IR experiment. The temperature of the sample cell was measured by a PT100 temperature sensor. In addition, magic-angle IR pump-probe experiment was carried out at room temperature in order to evaluate the vibrational lifetime of the amide-I mode of NEPA in D_2_O.

### Quantum chemistry calculations

Geometry optimization of NEPA and vibrational frequency analysis of its amide-I band, and those of NEPA-3(D_2_O) clusters (Scheme 1), were carried out using the density functional theory (DFT) at the level of B3LYP/6-311++G (d, p). To simulate the second and weak hydrogen bond, the distance between the oxygen of the amide unit and that of the second water was fixed at 4.0 Å during the optimization. The energies of the *trans* NEPA and *cis* NEPA were obtained at the level of M062X/def2TZVP. All calculations were performed using Gaussian 09 program^76^.

### Molecular dynamics simulations

MD simulations of NEPA in D_2_O was performed using the NAMD program^77^ with CHARMM force field^78^ for NEPA. A SPC/E model was used for water. One NEPA molecule was solvated in a cubic solvent box with an initial size of 28 × 28 × 28 Å^3^ containing 659 water molecules. The nonbonded cutoff distance was set to 12 Å, and the particle mesh Ewald summation was used for long-range electrostatic interaction. Before the MD simulations, the equilibration run was performed to ensure a stable NPT ensemble. The MD simulations were finally performed using the Langevin-piston Nose-Hoover method for 5 ns with a step of 20 fs using the NPT ensemble at normal atmospheric pressure and at 298 K. In discussing the MD results and hydrogen bond dynamics, H and D are used interchangeably in this work.

### NMR spectroscopy

NMR sample was prepared by dissolving NEPA in H_2_O at 200 mM concentration. One-dimensional ^1^H and ^13^C NMR spectra were measured using a 600 MHz Bruker Avance NMR spectrometer. The NMR results were shown in Supplementary Fig. 2 and the chemical shifts were listed in Supplementary Table 1. Chemical shifts of H, H1, H2/H4, and H3 of *trans* NEPA are about 7.85, 3.13, 1.05 and 2.16 ppm respectively. The number ratio of four types of H agrees with the number of different H atoms in NEPA, indicating no *cis* amide conformation for NEPA. Chemical shifts of C, C1, C2, C3, and C4 are about 176.94, 34.03, 13.15, 28.76, and 9.24 ppm respectively. The lack of no extra chemical shifts for the same type of C atoms for the *cis* NEPA also provides no evidence for the assignment of *cis* amide conformation.

### Supplementary information

Supplementary Material

Description of Additional Supplementary Files

Supplementary Data 1

## Data Availability

The data that support the findings of this study are available within the article and its Supplementary Information and Supplementary Data 1, or from the corresponding author upon reasonable request.
