# Supplementary material for: Chasing weakly-bound biological water in aqueous environment near the peptide backbone by ultrafast 2D infrared spectroscopy
Source: Commun Chem. 2024 Apr 11;7:82. doi: 10.1038/s42004-024-01170-x (PMC11009226; doi:10.1038/s42004-024-01170-x)
Supplement: Supplementary file 1 — Supplementary Material [file 42004_2024_1170_MOESM1_ESM.pdf]

Supplementary Information

Chasing weakly-bound biological water in aqueous environment near the peptide backbone by ultrafast 2D infrared spectroscopy

*Juan Zhao[1,2], Pengyun Yu[1,2], Tiantian Dong[1,2], Yanzhou Wu[1,2], Fan Yang[1,2], and Jianping Wang[1,2]**

1 Molecular Reaction Dynamics Laboratory, CAS Research/Education Center for Excellence in Molecular Sciences, Institute of Chemistry, Chinese Academy of Sciences, Beijing, 100190, P. R. China

2 University of Chinese Academy of Sciences, Beijing 100049, P. R. China

** Corresponding author. Tel: (+86)-010-62656806; Fax: (+86)-010-62563167; E-mail: jwang@iccas.ac.cn*

ORCID: Jianping Wang: 0000-0001-7127-869X.

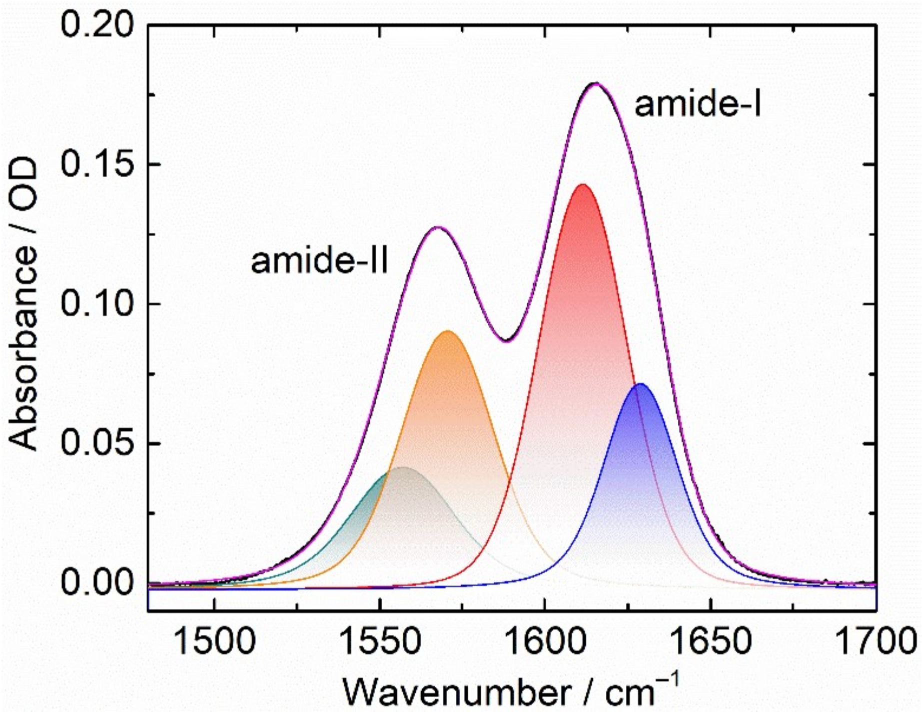

Supplementary Fig. 1: FTIR spectrum (black line) of non-deuterated NEPA in regular water (H_2O) obtained at 1 M concentration at 25 °C. The main amide-I peak is located at *ca.*1615.5 cm^{-1}, which can also be fitted by two Voigt functions. Two subbands (red filled and blue filled peaks) are peaked at 1611.5 and 1628.9 cm^{-1} respectively. The amide-II band which contains the N-H in-plane bending also appears in this frequency region because the sample is non-deuterated. Due to the presence of the SHB/WHB states of the amide C=O, the amide-II band also has two subbands, which are shown as a weak component on the low-frequency side and a strong component on the high-frequency side. Filled orange and green peaks are the fittings of the two sub bands of the amide-II mode, whose peak positions are 1557.1 and 1570.6 cm^{-1} respectively, with the integrated area ratio being 1:1.77. It is well known that the amide-I and II modes are anticorrelated in frequency, so the fitting shown in this figure is reasonable. The overall fitting of the amide-I and II bands is given (magenta line) and agrees well with the experimental result (black). For deuterated NEPA in D_2O, the amide-II mode is known to be red shifted by ca. 100 cm^{-1} and is out of the spectral window of this figure.

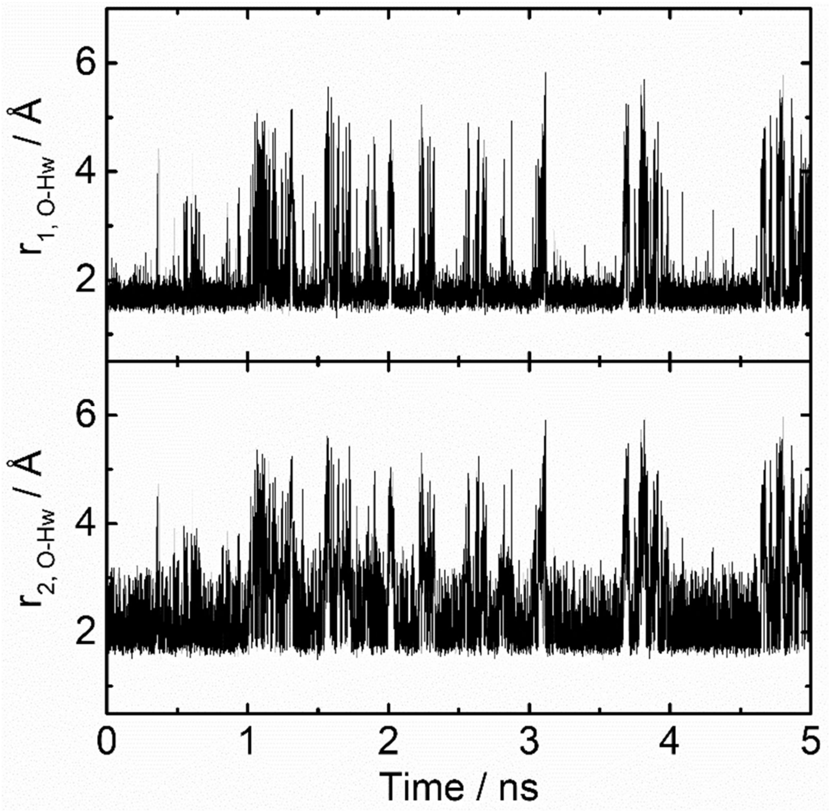

Supplementary Fig. 2: Time evolutions of the nearest ($r_{1,\text{ O-HW}}$) and the second near ($r_{2,\text{ O-HW}}$) distances between NEPA carbonyl O atom and water H atom.

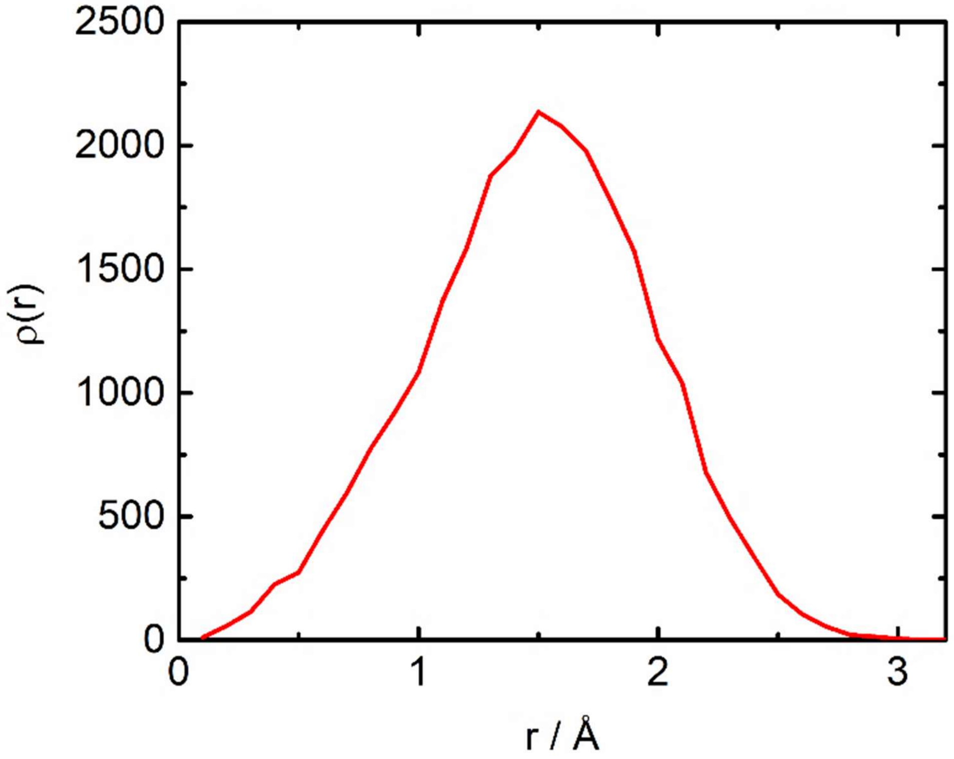

Supplementary Fig. 3: Probability of the nearest distance between the H atom of the NH group of NEPA and water O atom.

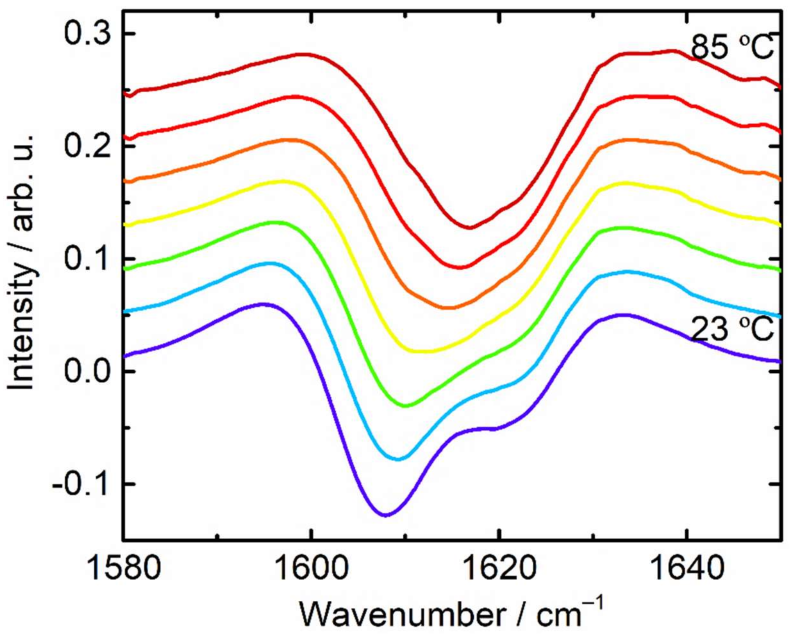

Supplementary Fig. 4: Second-derivative FTIR spectra of deuterated NEPA in D_2O in the amide-I region as a function of temperature (23, 35, 45, 55, 65, 75 and 85 °C).

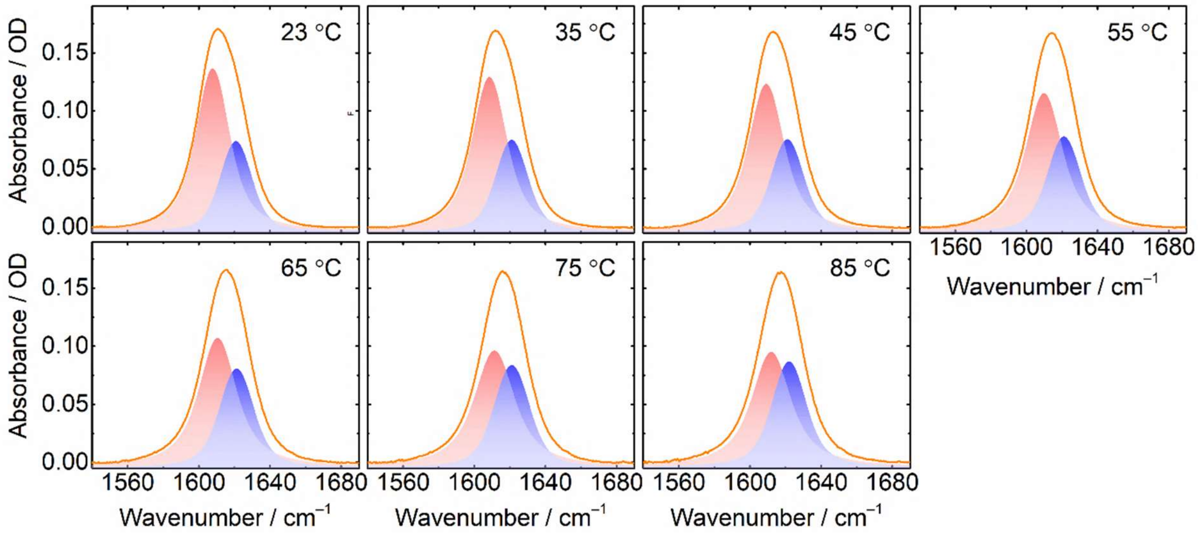

Supplementary Fig. 5: Infrared spectra of the amide-I band of deuterated NEPA in D_2O as a function of temperature. The orange line in each panel is the experimental result. In each case, two shaded peaks are the fitting results using Voigt function.

Supplementary Table 1: Peak frequency (ω in cm^{-1}), relative peak areas and their ratio (A_{SHB}/A_{WHB}) of the two absorption components obtained by fitting the amide-I band of NEPA at different temperatures (T in °C). Equilibrium constant (K_{eq}) is computed at each temperature using Eq. (1). Values in parentheses is standard deviation obtained by fitting.

T	ω_{SHB}	ω_{WHB}	A_{SHB}	A_{WHB}	A_{SHB}/A_{WHB}	K_{eq}
23	1607.9	1621.0	4.5156 (0.0109)	2.0536 (0.0117)	2.199 (0.0136)	0.5214
35	1608.6	1621.0	4.4552 (0.0125)	2.1816 (0.0130)	2.042 (0.0135)	0.5614
45	1609.3	1621.2	4.4341 (0.0145)	2.2960 (0.0147)	1.931 (0.0139)	0.5937
55	1610.0	1621.2	4.3441 (0.0212)	2.4043 (0.0211)	1.807 (0.0181)	0.6345
65	1610.7	1621. 4	4.2292 (0.0310)	2.5370 (0.0318)	1.667 (0.0242)	0.6878
75	1611.4	1621.1	4.0534 (0.0951)	2.6435 (0.0999)	1.533 (0.0682)	0.7477
85	1612.1	1622.2	4.0005 (0.0523)	2.7565 (0.0531)	1.451 (0.0338)	0.7900

Supplementary Note 1: The standard deviation of the peak area was obtained from the fitting result in Supplementary Fig. 6, and that of the area ratio and the equilibrium constant (K_{eq}, Fig. 2) was obtained by an error propagation formula, which is expressed as following: for $w = x/y$, $\sigma_w/w = \sqrt{(\sigma_x/x)^2+(\sigma_y/y)^2}$, where σ_x and σ_y are the standard deviation of data x and y, respectively, and σ_w is the standard deviation of data w. Further, the following formula is used in plot Fig. 2b: for $w = ln(x)$, $\sigma_w = \sigma_x/x$, where σ_x is the standard deviation of data x, and σ_w is the standard deviation of data w.

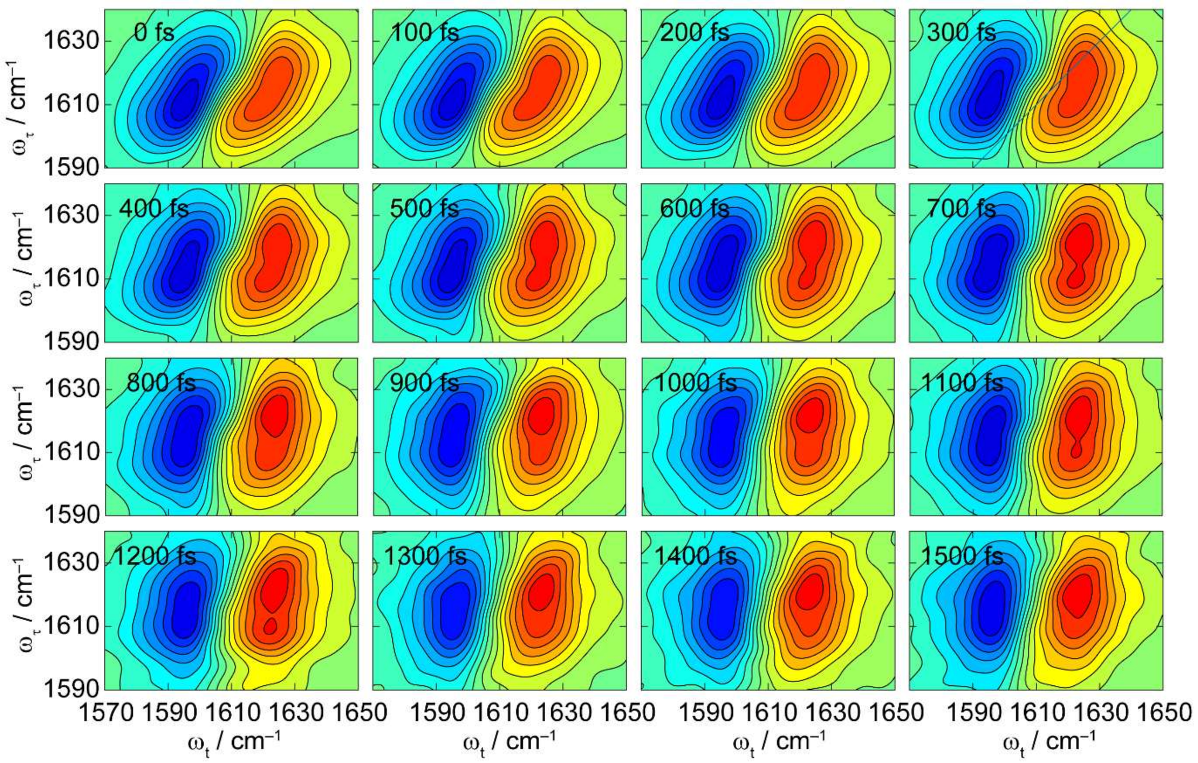

Supplementary Fig. 6: Purely absorptive 2D IR spectra as a function of the waiting time (T_w) at 23 °C.

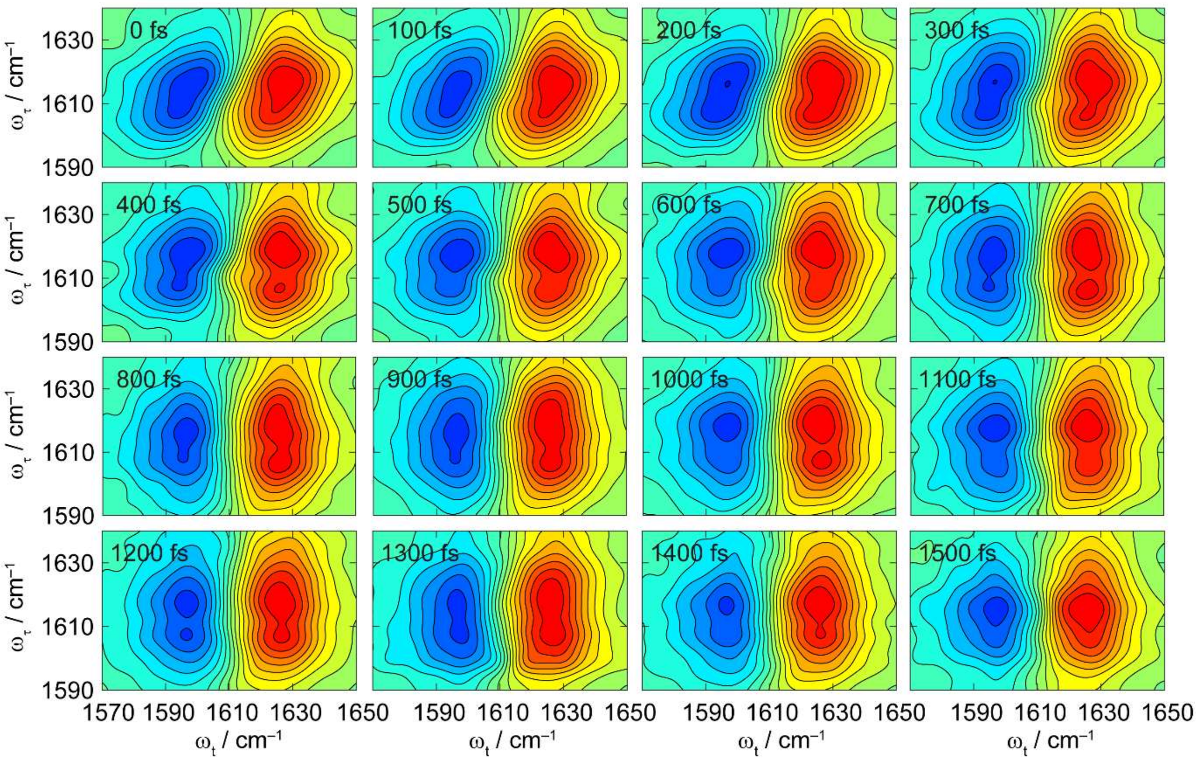

Supplementary Fig. 7: Purely absorptive 2D IR spectra as a function of the T_w at 50 °C.

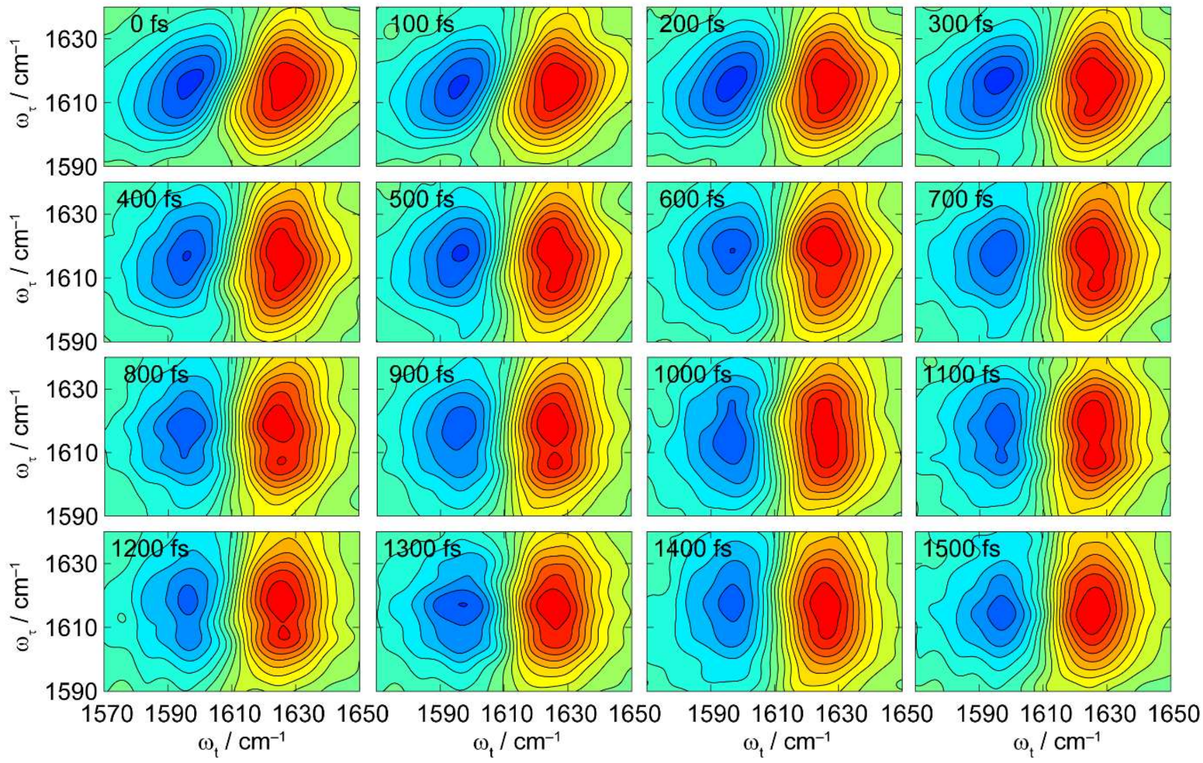

Supplementary Fig. 8: Purely absorptive 2D IR spectra as a function of the T_w at 60 °C.

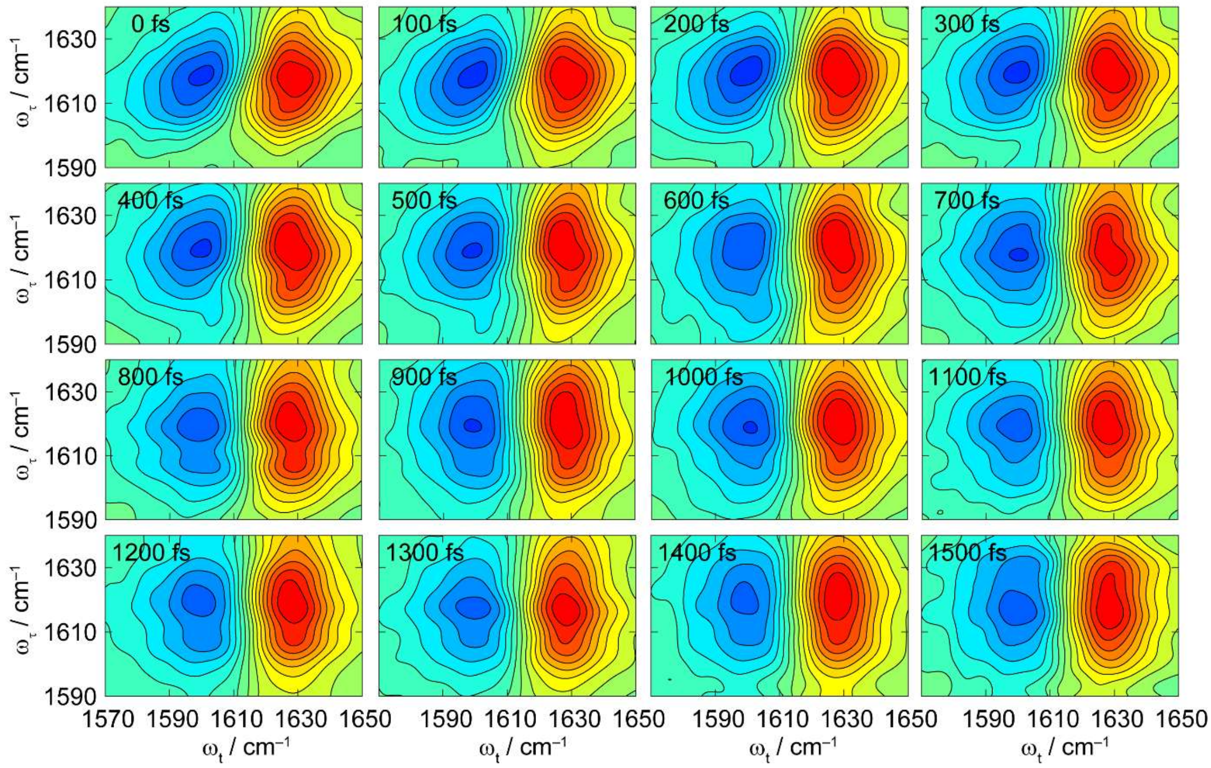

Supplementary Fig. 9: Purely absorptive 2D IR spectra as a function of the T_w at 85 °C.

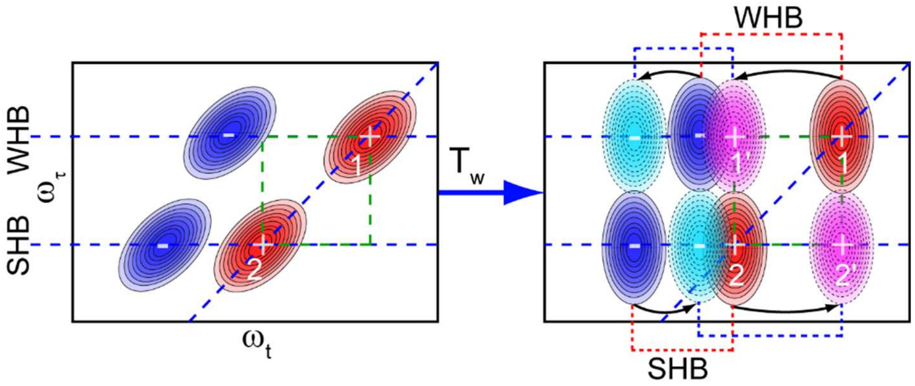

Supplementary Fig. 10: An illustration of a typical 2D IR spectrum of the amide-I mode of NEPA at the initial moment and longer T_w in the presence of chemical exchange. Two pairs of diagonal peaks are shown (left), where peak 1 and 2 (red) represent the high- and low-frequency components (i.e., the WHB and SHB states), coming from corresponding 0-1 transition (i.e., vibrational transition from $v = 0$ to $v = 1$, which is a positive signal). Chemical exchange occurs as T_w increases, yielding two pairs of off-diagonal peaks, where peak 1' is the 0-1 transition of the chemical exchange-induced low-frequency component, whereas peak 2' is the 0-1 transition of the exchange-induced high-frequency component. In reality spectral overlap between positive and negative signals occurs so that the apparent spectral intensity in the area of peak 2 (and that of peak 1′) is mostly affected.

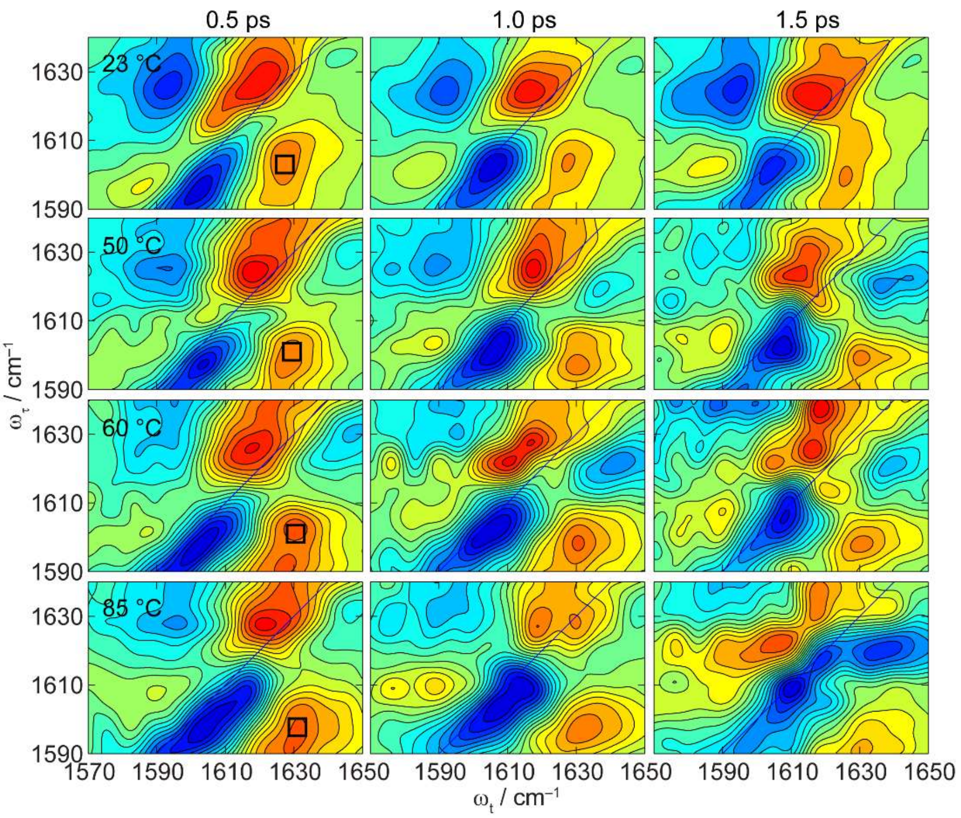

Supplementary Fig. 11: Illustration the presence of off-diagonal peaks due to chemical exchange at three typical waiting times (T_w) by subtracting the 2D spectrum at $T_w = 0$ ps from those at varying waiting times, with black squares at 0.5 ps showing the location of an integrated area for off-diagonal signal (23 °C: ω_τ = 1601 - 1606 cm^{-1} and ω_t = 1625 - 1630 cm^{-1}; 50 °C: ω_τ = 1599 - 1604 cm^{-1} and ω_t = 1627 - 1632 cm^{-1}; 60 °C: ω_τ = 1599 - 1604 cm^{-1} and ω_t = 1628 - 1633 cm^{-1}; 85 °C: ω_τ = 1596 - 1601 cm^{-1} and ω_t = 1629 - 1634 cm^{-1}). However, the cross peaks were directly taken from Supplementary Figs. 7-10 for kinetics analysis as a function of T_w shown in Fig. 4b. Note that the ω_t value of the selected region slightly shifts to the higher-frequency side as a function of temperature, agreeing with the results shown in Supplementary Fig. 6 and Table 2.

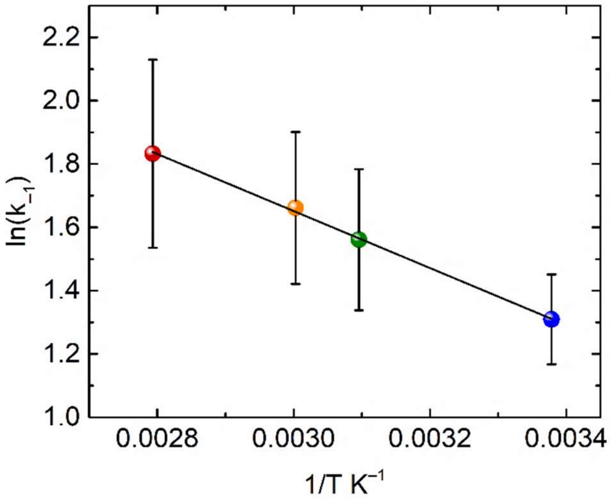

Supplementary Fig. 12: Arrhenius plot of the rate constants k_{-1} for the change from the WHB state to the SHB state versus temperature and its fitting (black). The rate constant k_{-1} was obtained from the follow equation, $k_1 = k_{-1}e^{-\Delta G/k_B T}$. If the term $e^{-\Delta G/k_B T}$ is taken as a constant at each temperature (i.e., ignoring the uncertainty of ΔG for simplification), then the error of k_{-1} can be acquired according to the following error propagation formula: for $y = ax$, where a is a constant, $\sigma_y = |a|\sigma_x$. σ_x is the standard deviation of data x, and σ_y is the standard deviation of data y.

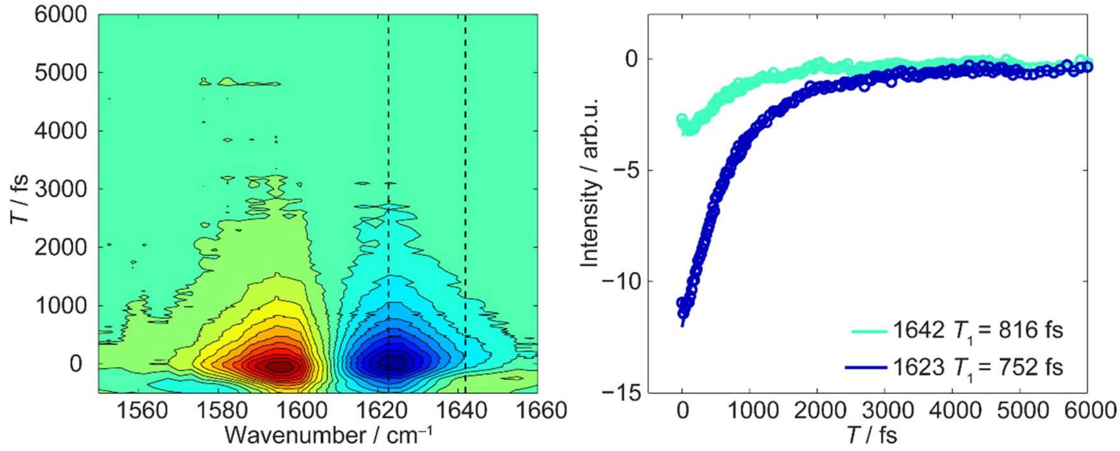

Supplementary Fig. 13: Magic-angle IR pump-probe spectra of the amide-I mode of deuterated NEPA in D_2O at 23 °C (left) and the vibrational relaxation dynamics traces extracted from the spectra (right) as a function of pump-probe delay time (T). Results at two probing frequency positions (1642 and 1623 cm^{-1}) provide a reasonable estimation of the vibrational relaxation time (T_1) for the WHB and SHB states respectively.
