# Supplementary material for: Chasing weakly-bound biological water in aqueous environment near the peptide backbone by ultrafast 2D infrared spectroscopy
Source: Commun Chem. 2024 Apr 11;7:82. doi: 10.1038/s42004-024-01170-x (PMC11009226; doi:10.1038/s42004-024-01170-x)
Supplement: Supplementary file 2 — Description of Additional Supplementary Files [file 42004_2024_1170_MOESM2_ESM.pdf]

Description of Additional Supplementary Files

File name: Supplementary Data 1

Description: ^{1}H and ^{13}C NMR spectra of NEPA
