# Supplementary material for: Chasing weakly-bound biological water in aqueous environment near the peptide backbone by ultrafast 2D infrared spectroscopy
Source: Commun Chem. 2024 Apr 11;7:82. doi: 10.1038/s42004-024-01170-x (PMC11009226; doi:10.1038/s42004-024-01170-x)
Supplement: Supplementary file 3 — Supplementary Data 1 [file 42004_2024_1170_MOESM3_ESM.pdf]

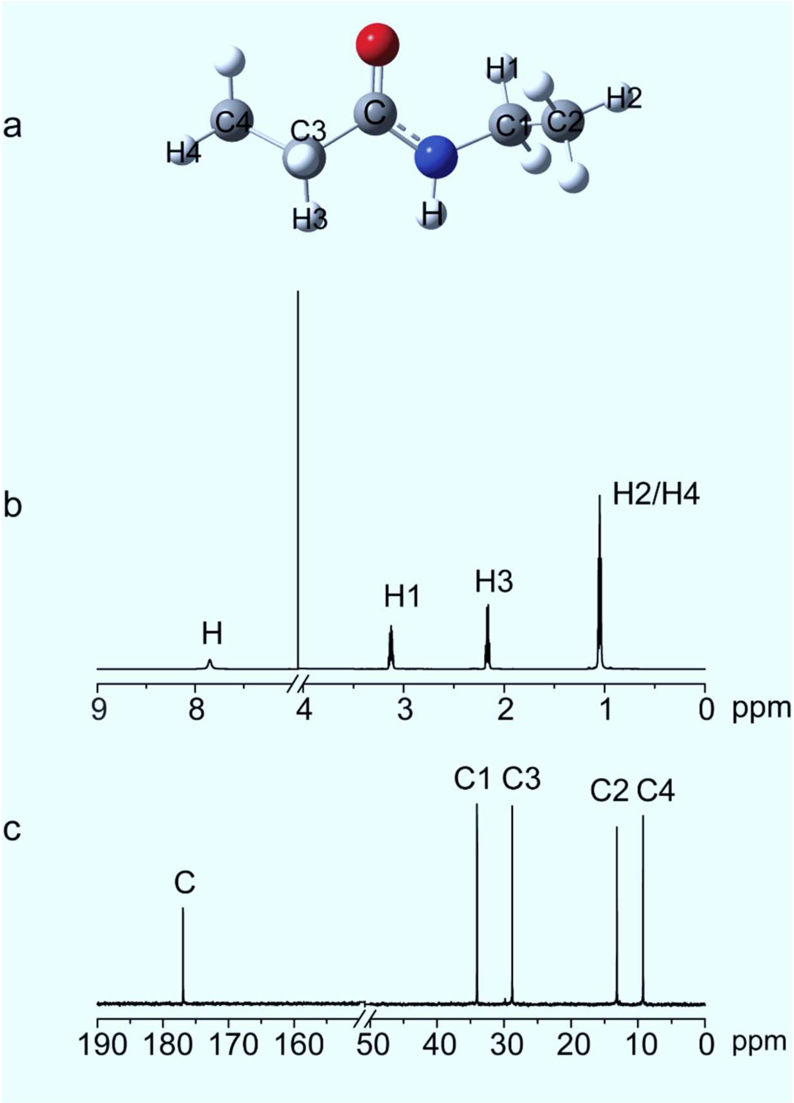

Supplementary Data 1: Structure of NEPA (a) and 600-MHz ^{1}H (b) and ^{13}C (c) NMR spectra of NEPA in H_2O at 23 °C, with ^{1}H and ^{13}C chemical shifts (δ, in ppm) given below.

Atom type	δ	Atom type	δ
H	7.85	C	176.94
H1	3.13	C1	34.03
H2/H4	1.05	C2	13.15
H3	2.16	C3	28.76
		C4	9.24
